# Antibody Responses against Xenotropic Murine Leukemia Virus-Related Virus Envelope in a Murine Model

**DOI:** 10.1371/journal.pone.0018272

**Published:** 2011-04-06

**Authors:** Natalia Makarova, Chunxia Zhao, Yuanyuan Zhang, Sushma Bhosle, Suganthi Suppiah, Jeanne M. Rhea, Natalia Kozyr, Rebecca S. Arnold, Hinh Ly, Ross J. Molinaro, Tristram G. Parslow, Eric Hunter, Dennis Liotta, John Petros, Jerry L. Blackwell

**Affiliations:** 1 Emory Vaccine Center, Emory University, Atlanta, Georgia, United States of America; 2 Yerkes National Primate Research Center, Emory University, Atlanta, Georgia, United States of America; 3 Division of Infectious Diseases, Emory University, Atlanta, Georgia, United States of America; 4 Department of Pathology and Laboratory Medicine, Emory University, Atlanta, Georgia, United States of America; 5 Department of Urology, Emory University, Atlanta, Georgia, United States of America; 6 Department of Hematology-Oncology, Emory University, Atlanta, Georgia, United States of America; 7 Department of Chemistry, Emory University, Atlanta, Georgia, United States of America; 8 Core Laboratories Emory University Hospital Midtown, Emory University, Atlanta, Georgia, United States of America; 9 Atlanta Veterans Affairs Medical Center, Decatur, Georgia, United States of America; University of Pittsburgh, United States of America

## Abstract

**Background:**

Xenotropic murine leukemia virus-related virus (XMRV) was recently discovered to be the first human gammaretrovirus that is associated with chronic fatigue syndrome and prostate cancer (PC). Although a mechanism for XMRV carcinogenesis is yet to be established, this virus belongs to the family of gammaretroviruses well known for their ability to induce cancer in the infected hosts. Since its original identification XMRV has been detected in several independent investigations; however, at this time significant controversy remains regarding reports of XMRV detection/prevalence in other cohorts and cell type/tissue distribution. The potential risk of human infection, coupled with the lack of knowledge about the basic biology of XMRV, warrants further research, including investigation of adaptive immune responses. To study immunogenicity *in vivo*, we vaccinated mice with a combination of recombinant vectors expressing codon-optimized sequences of XMRV *gag* and *env* genes and virus-like particles (VLP) that had the size and morphology of live infectious XMRV.

**Results:**

Immunization elicited Env-specific binding and neutralizing antibodies (NAb) against XMRV in mice. The peak titers for ELISA-binding antibodies and NAb were 1∶1024 and 1∶464, respectively; however, high ELISA-binding and NAb titers were not sustained and persisted for less than three weeks after immunizations.

**Conclusions:**

Vaccine-induced XMRV Env antibody titers were transiently high, but their duration was short. The relatively rapid diminution in antibody levels may in part explain the differing prevalences reported for XMRV in various prostate cancer and chronic fatigue syndrome cohorts. The low level of immunogenicity observed in the present study may be characteristic of a natural XMRV infection in humans.

## Introduction

Xenotropic murine leukemia virus-related virus (XMRV) was first identified through microarray analysis of human prostate cancer (PC) samples from patients with an inherited defect in RNASEL (R462Q variant), a downstream effector of the antiviral interferon defense pathway [1], [2]. The presence of gammaretroviral genomes was further confirmed by *gag*-specific nested RT-PCR and FISH [2]. Based on sequence analysis, XMRV is closely related to mouse exogenous gammaretroviruses that are known to cause leukemias and lymphomas in different host species. Since its original identification, XMVR has been detected in several independent investigations. In one study XMRV was isolated from the prostate carcinoma cell line 22Rv1 [3]. Multiple XMRV chromosomal integration sites were found in the 22Rv1 cell line as well as in that of cancer tissues of PC patients [4]. Although it does not have common integration sites within or near proto-oncogenes or tumor suppressor genes [3], XMRV shows preferences for integration near cancer breakpoints, common fragile sites and microRNA [4]. Additional evidence for XMRV came from a study that analyzed a large cohort of patients with different stages of PC as well as healthy men, which revealed the prevalence of XMRV in malignant epithelial cells and an association with more aggressive tumors [5]. This study expanded the population of PC patients infected with XMRV to include those with normal RNASEL. Moreover, our recent publication further demonstrated the prevalence of XMRV in prostate tissue derived from an independent cohort of PC patients [6]. This study showed concordance between the presence of neutralizing antibodies (NAb) and XMRV nucleic acids detected by nested PCR and FISH. Another independent study has shown that XMRV is detectable in normal and tumor prostate tissue from patients with PC from the southern United States [7]. In addition to being identified in PC samples, evidence for XMRV was also found in a study of subjects with Chronic Fatigue Syndrome (CFS) that revealed the presence of XMRV in activated human B and T cells as well as detectable levels of anti-XMRV Env antibodies in nine out of 18 CFS human plasma samples [8]. In another recent study, a second related polytropic MLV-like virus was detected in separate cohort of 37 CFS subjects [9]. Collectively these studies provide evidence for infection of humans by these newly identified viruses that belong to a family of viruses that cause significant pathogenesis in their natural hosts [2], [5].

In contrast to the studies mentioned above, XMRV was not found in PC and CFS patient cohorts from several European and US studies. Studies of the prevalence of XMRV in two PC patient cohorts in Germany found, for example, no link between prostate cancer and the presence of XMRV when DNA or RNA from tumor samples was analyzed [10], [11]. Also, analyses of CFS cohorts from England and Netherlands failed to detect XMRV using PCR analysis [12], [13], [14]. Likewise, an ELISA-based screen of antibodies in plasma of PC patients detected no XMRV-specific responses [11] and no antibodies against XMRV were found in sera of CFS patients when XMRV pseudoviruses were used in a neutralization assay [12]. In a study from the Centers for Disease Control and Prevention (CDC), there was no evidence of XMRV infection in 50 CFS patients or 56 healthy controls [15]. Some have speculated that geographical restrictions account for the differences in detecting XMRV; however, the fact that the assays and reagents varied among the studies described above may also have contributed to the differences in findings. Thus, additional investigations are needed to sort out those discrepancies and reveal the true prevalence of XMRV infection.

In our recent study of XMRV serological prevalence in a cohort of PC patients, we observed approximately 25% positivity for serum XMRV antibodies [6]; however, despite this relatively high incidence, the XMRV antibody titers were low overall compared to those of HIV-1 infected individuals [16], [17]. To provide an explanation for the low magnitude of immune responses observed in our PC cohort, we initiated a study of XMRV immune responses in a murine model. We hypothesized that low immunogenicity is an inherent characteristic of an XMRV infection. To test this hypothesis, we vaccinated mice to elucidate the magnitude and duration of the antibody response against the XMRV Env antigen.

## Results and Discussion

### XMRV pseudovirus and NAb assay

**Figure 1:**
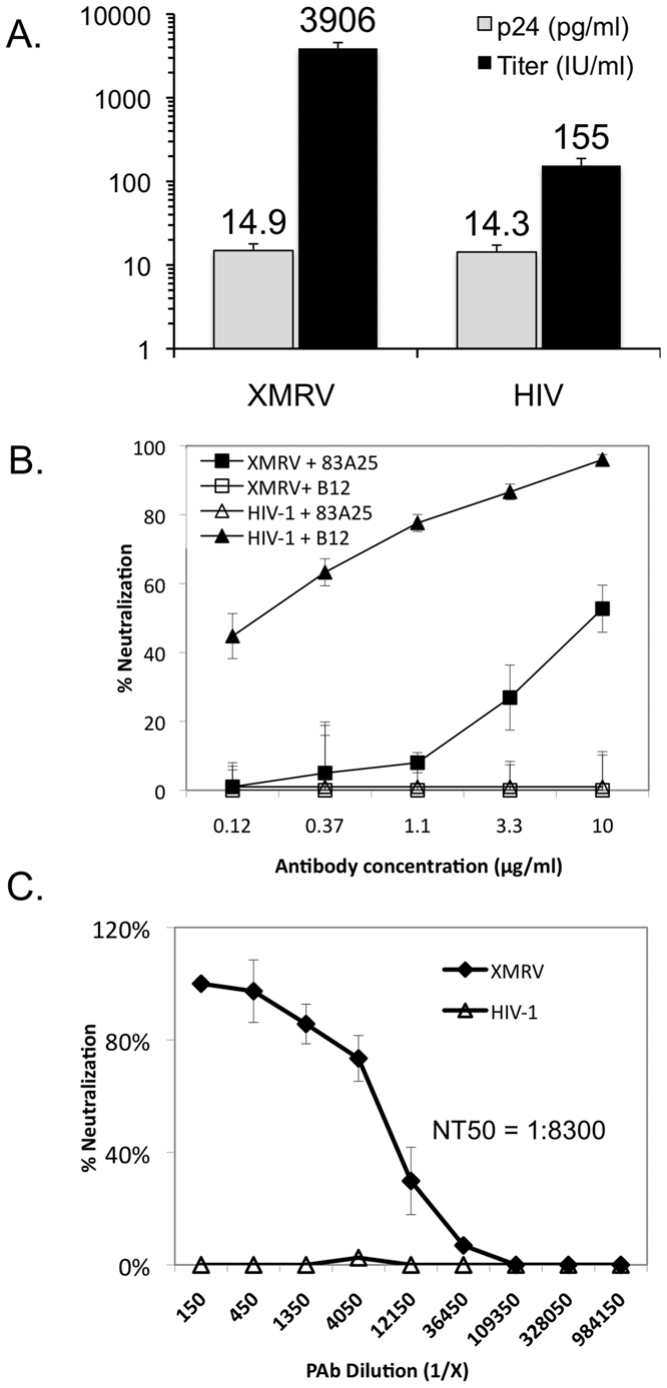
Characterization of XMRV pseudovirus and single-round neutralization assay. (A) Comparison of XMRV and control HIV-1 pseudoviruses in yield (p24 accumulation) and infectivity (IU/ml on TZM-bl cells). (B) Detection of antibody specificity to XMRV and HIV-1 pseudoviruses. Pseudoviruses were tested in the neutralization assay with mAb 83A25 that recognizes a shared epitope of MLV Env glycoprotein and with mAb b12 that recognizes HIV-1 Env glycoprotein. (C) Neutralization of the XMRV and HIV-1 pseudoviruses showing a broad range of sensitivity and specificity of the assay using polyclonal antibodies (anti Friend-MuLV).

An HIV-1 pseudovirus-based assay has been widely used for the detection of NAb in sera from HIV-1 infected patients and experimentally infected/vaccinated animal models [18], [19]. We therefore adapted the assay using an XMRV pseudovirus to determine the utility of such an approach for detecting XMRV NAbs. The infectivities of the XMRV and control HIV-1 pseudoviruses were compared by monitoring the levels of β-galactosidase expression in TZM-BL cells after 48 hours of infection (Figure 1A, black columns). The results indicated that the XMRV pseudovirus is ∼250 times more infectious than the control HIV-1 pseudovirus. The difference in infectivity between the two pseudoviruses was not due to *de novo* virus production, since the p24 protein compositions of the XMRV and HIV-1 pseudoviruses were the same (Figure 1A, grey columns). It is likely that the difference in infectivity is due to the codon-optimization algorithm that was used to synthesize the XMRV *env* gene, whereas the HIV-1 *env* gene used in this experiment was not codon-optimized. We next determined whether the XMRV pseudovirus could be employed in a NAb assay using monoclonal antibodies (mAb) b12 and 83A25 (Figure 1B). The mAb b12, which interacts with the CD4-binding site on the HIV-1 Env glycoprotein, efficiently neutralized the HIV-1 pseudovirus but did not neutralize the XMRV pseudovirus. Conversely, mAb 83A25, which has been shown to neutralize several related MuLV strains [20], inhibited infection of the XMRV pseudovirus in a dose-dependent manner, but had no effect on the infectivity of HIV-1 pseudovirus. We then compared the XMRV and HIV-1 pseudoviruses in the NAb assay using polyclonal antibodies (PAb) produced against Friend MuLV virus. The PAb neutralized the XMRV pseudovirus over a wide dilution range, but did not inhibit the HIV-1 pseudovirus at any dilution (Fig. 1C). The neutralizing antibody titer that reduced XMRV infection by 50% (NT50) was ∼1∶8300. Collectively, these data demonstrate that (1) the XMRV Env can be pseudotyped onto HIV-1 viral particles and that these XMRV pseudoviruses can (2) efficiently infect the reporter cell line TZM-BL and (3) be used to detect XMRV-specific antibodies with specificity and sensitivity over a wide range of dilutions.

### Characterization of XMRV expression vectors

**Figure 2:**
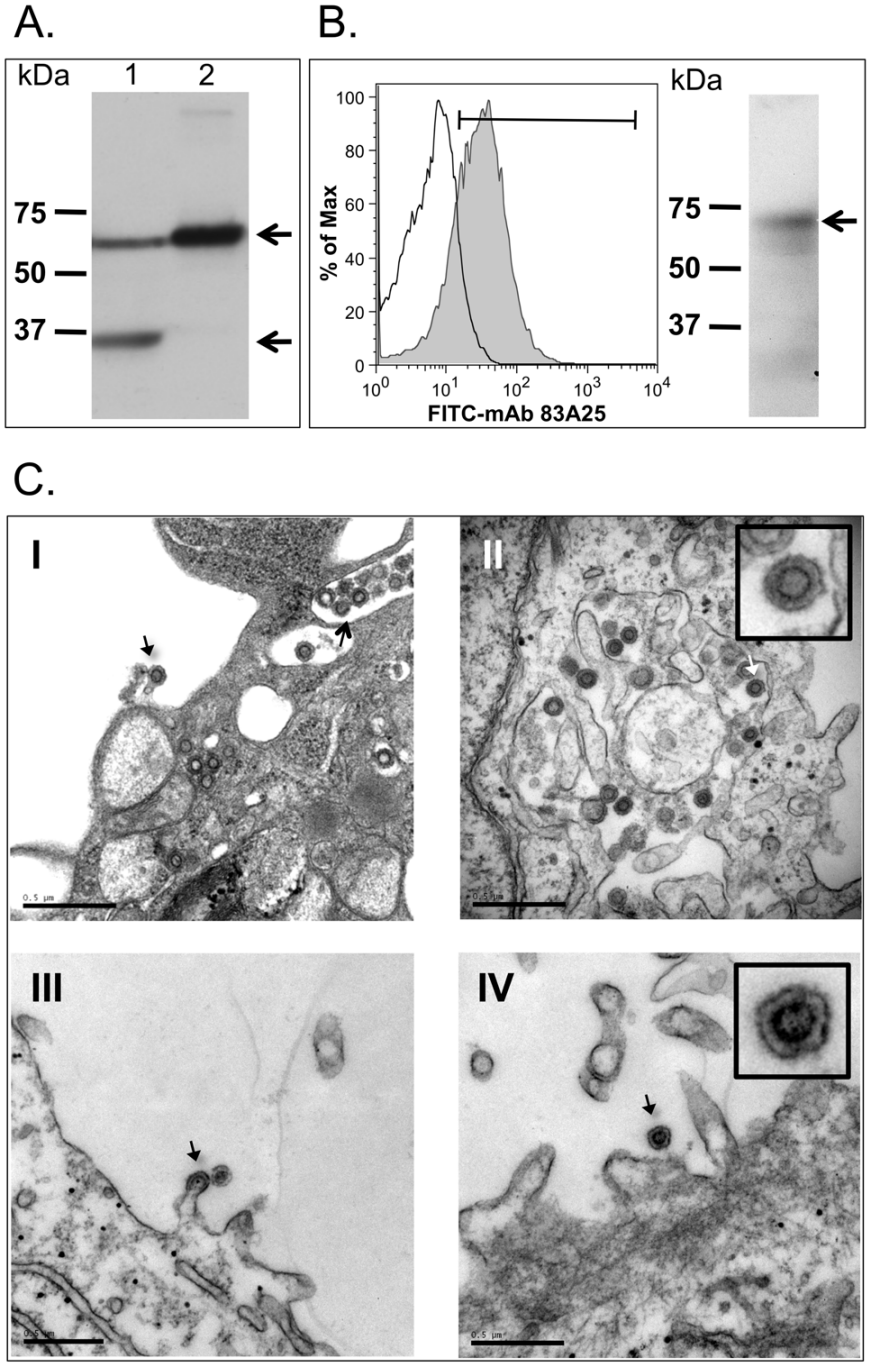
Expression of XMRV Env, Gag and VLP. (A) Western blot analysis of XMRV gag expression. HeLa cells were infected with Ad5-XMRV (10 MOI) for 24 h and then whole cell lysate (Lane 1) and cell culture media concentrated 100-fold by centrifugation through a 20% sucrose cushion (Lane 2) were subjected to 10% SDS-PAGE and then transferred to PVDF. The blots were probed with anti-Gag mAb R187 and HRP-conjugated goat anti-rat immunoglobulin G antiserum (Southern Biotechnology Associates, Inc.). The masses (kDa) of the molecular weight standards (Std) are shown on the left. The arrows (←) indicate the positions of the Gag precursor at ∼65 kDa (top arrow) and a cleaved, lower molecular mass Gag protein (bottom arrow). (B) Detection of XMRV envelope expression by flow cytometric (left) and Western blot (right) analyses. For flow cytometry, HeLa cells infected as in (A) were stained with mAb 83A25 and fluorescein isothiocyanate-conjugated goat anti-rat immunoglobulin G antiserum. For Western blot analysis, VLP produced by those cells were purified from culture media and probed with mAb 83A25. MAb 83A25 recognizes an epitope located near the carboxyl terminus of Env that common for many MuLVs. (C) Electron microscopy showing VLP production in HeLa cells after 48 hours of infection with Ad5-XMRV (Panels I and II). An infectious XMRV virus is shown budding (arrows) from Du145-C7 cells, a prostate cancer cell line that constitutively produces XMRV (Panels III and IV). The similarities in morphology and size between the VLP and live XMRV particles are in the insets of Panels II and IV.

To study XMRV immunogenicity in a mouse model, we next generated plasmid and recombinant Ad5 vectors, called pDP1-XMRV*envgag* and Ad5-XMRV, respectively, that co-express the XMRV *gag* and *env* genes. XMRV *gag* gene product expression was determined by infecting HeLa cells with Ad5-XMRV, followed by a Western blot analysis using mAb R187 [2], which showed the Gag precursor at ∼65 kDa (Lane 1, top arrow) and a cleaved lower molecular mass Gag protein (Lane 1, bottom arrow) in the cytosolic lysate (Fig. 2A). The latter is likely to be a product of non-specific cleavage by host proteases, since the viral protease was not expressed. Only the immature Gag protein was detected after pelleting the media through a sucrose cushion (Lane 2), since VLP do not contain virus specific proteases that are required for Gag maturation. We also detected XMRV Env expression using mAb 83A25. Flow cytometric analysis of HeLa cells infected with Ad5-XMRV detected surface and intracellular XMRV Env expression (Fig. 2B left). The presence of XMRV Env in purified virus-like particles (VLP) was indicated by Western blot analysis. (Fig. 2B right).

It was shown previously that the infection of cells with Ad5 vectors that co-express HIV-1 *gag* and *env* genes leads to the production of virus-like particles (VLP) [21]. Our XMRV VLP are different from the virus in that they are not infectious since infectivity requires Gag protein processing and virus maturation. However, the Env protein is folded and exposed on the VLP in the same way it is present on native virus. In this regard, using transmission electron microscopy (TEM) we detected XMRV VLP in HeLa cells infected with Ad5-XMRV (Fig. 2C, Panels I and II). XMRV VLP budding was observed (Fig. 2C, Panel I) that was comparable to virus budding from DU145-C7 cells that produce infectious XMRV (Fig. 2C, Panels III and IV). We also observed an accumulation of XMRV VLP in intracellular vesicles (Fig. 2C, Panel I) and their dispersal throughout the cytosol of the cells infected with Ad5-XMRV (Fig. 2C, Panel II). This observation suggests that VLP may also assemble within multivesicular bodies (MVP) in which case Env is recycled from the plasma membrane and then interacts with Gag on the MVP membrane [22]. The XMRV VLP were similar in size and morphology (see Panel II and IV insets) to those observed in the culture media of 22Rv1 cells [3] and DU145 cells transfected with a full-length XMRV molecular clone [5]. Based on our data *in vitro*, we predict that XMRV VLP production occurs in cells infected with Ad5-XMRV *in vivo* after immunization.

### Immunization of mice and detection of neutralizing sera against XMRV

**Figure 3:**
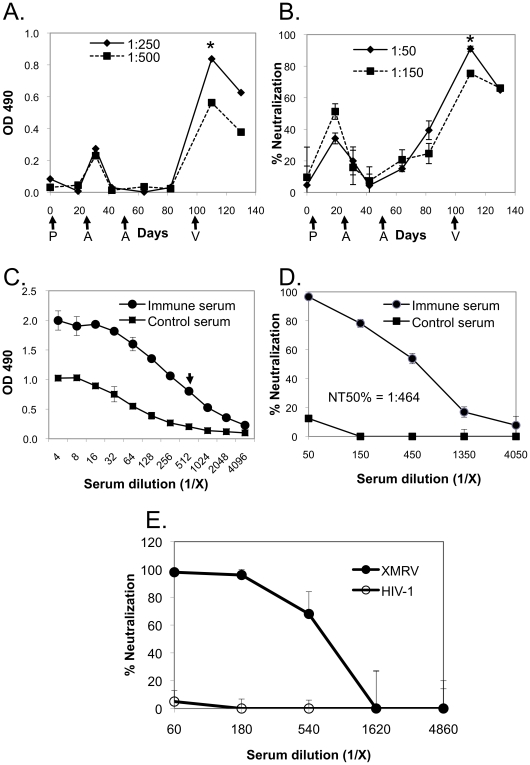
Detection of XMRV-specific antibody production in mouse sera. Time course of the production of (A) ELISA-binding antibodies and (B) NAb in Balb/C mice (10 animals in each group) immunized with pDP1-XMRV*envgag* (first arrow; P), Ad5-XMRV (second and third arrows; A) and XMRV VLP (fourth arrow; V). Determination of (C) endpoint dilution and (D) serum neutralizing titers at the peak time point indicated by asterisks in Panels A and B, respectively. The arrow indicates endpoint dilution. (E) The specificity of the serum neutralizing activity was determined by comparing XMRV and HIV-1 pseudoviruses and showed that the primary target for neutralization is the XMRV Env.

We next sought to determine whether immunization with the XMRV VLP-expressing vectors would elicit an anti-Env antibody response. Such VLP-based vaccinations against other viruses have been efficacious [23], [24], [25], [26] and may be important when the antigenicity and immunogenicity of the Env protein are affected by the structural context of the epitope(s) [27]. We used the immunization scheme of DNA priming and Ad5 boosting that has been successfully applied in multiple systems [28], [29], [30], [31], [32]. The Ad5 vector has been well characterized for efficient vaccine delivery and is well suited for the co-delivery of multiple antigens into the same cells. In addition, Ad5 vectors activate the innate immune system initiating the production of pro-inflammatory cytokines and differentiation of immature dendritic cells into professional antigen-presenting cells [32]. Thus, we expected that our DNA prime/Ad5 boost regiment would stimulate XMRV-specific immune responses. To determine the immunogenicity of XMRV Env, Balb/C mice (10 animals per group) were primed with pDP1-XMRV*envgag* plasmid on Day 0, and then boosted with recombinant Ad5-XMRV at 22 and 50 days after priming. Binding antibodies were detected after the first Ad5 boost then declined to baseline within 20 days and were not boosted by a second Ad5 immunization (Fig. 3A). XMRV NAb were detected after the plasmid DNA prime (Fig. 3B); however, they dropped to nearly undetectable levels in 20 days and were not boosted by the first Ad5-XMRV vaccination. The second Ad5-XMRV boost modestly increased the NAb activity by ∼20%. The mice were then boosted once again on Day 100 with XMRV VLP (7.5 µg per mouse). The resulting binding antibodies and NAb were increased 6- and 3-fold, respectively, but started to decrease (>20%) again by the end of the experiment on Day 130 (Fig. 3A–B).

We next determined the titers of the serum samples collected at peak (i.e., at 10 days after the XMRV VLP boost). The ELISA end-point dilution titer for the immune sera was 1∶512 (Fig. 3C) and the NAb titer that inhibited infection by 50% (NT50) was 1∶464 (Fig. 3D). The specificity of immune serum was further assessed in a NAb assay using control HIV-1 pseudovirus (Fig. 3E). Immune sera did not neutralize the negative control HIV-1 pseudovirus, but did neutralize the XMRV pseudovirus with high efficiency at up to 1∶540 dilution. It is important to note that the only difference between these XMRV and HIV-1 pseudoviruses is the Env protein; therefore, neutralizing activity detected in the immune serum is primarily directed against XMRV Env rather than against host cell proteins incorporated into the lipid membrane, which can be major antibody targets when using VLP- or virion-based immunogens produced in host cells from a different species [33].

### Immunization elicits XMRV NAb

**Figure 4:**
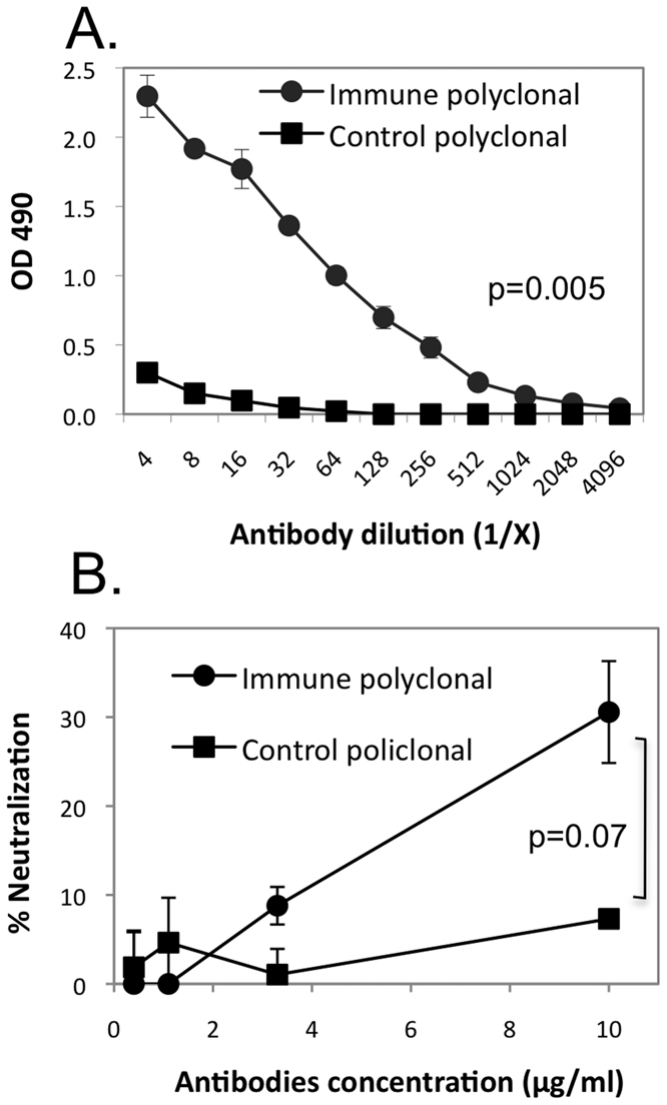
Characterization of antibodies purified from immune and control mouse sera. Total immunoglobulin pool was affinity purified from immune or control sera collected at the peak time point of neutralizing activity (figure 3B). The ELISA-binding (A) and NAb (B) activities were then measured as described in Figure 3 and showed that immunization elicited an immune response leading to the production of anti-XMRV immunoglobulins.

We next purified total immunoglobulin (Ig) from the immune and control sera to further characterize the serum neutralizing activity. Similar to the results using sera, significant binding activity was detected with purified Ig from the XMRV-immunized group as compared to the control group (Fig. 4A). In addition, we observed significant NAb activity in the XMRV-immunized animals at the 10 µg/ml concentration as compared to the same concentration in the control group (Fig. 4B). Thus, delivery of XMRV antigens clearly elicits a humoral immune response in mice that leads to the production of XMRV-specific binding antibodies and NAb.

Although we were able to elicit XMRV Env antibodies, the magnitude of the response was lower than that observed following immunization with other retrovirus VLP [34]. Though speculative at this point, there are several possibilities to explain this result. One explanation is that glycosylation of the Env proteins could conceal some of the antigenic sites necessary for the host to mount a robust neutralizing immune response, as this is known to occur with other retroviruses [35]. It is also possible that partial tolerance due to the presence of endogenous murine retroviruses may have diminished the immunogenicity of the XMRV Env protein in the mouse model that we used here. In this regard, it has been shown that human and murine endogenous retroviruses can account for the lack of immunogenicity of some tumor-associated viral antigens [36]. To test this possibility we are currently investigating XMRV immunity in rabbit and non-human primate models. Another possibility is that the XMRV Env has immunosuppressive activity that reduces its immunogenicity, which has been shown with related murine and primate Env proteins [37], [38], [39]. With these considerations in mind, ongoing studies are underway to optimize the immunization regime.

Discrepancies among current reports on XMRV prevalence, and gaps in what is known about its role in transformation, transmission and pathogenesis, provide an impetus for basic investigation of XMRV and the development of standardized detection assays. We undertook the present study in order to determine the immunogenicity of the XMRV Env in an experimental model and, in the process, developed ELISA and NAb assays for measuring anti-XMRV immunity. Here we demonstrate that the XMRV Env protein is immunogenic in a mouse model but that the resulting antibody responses are low in magnitude and short in duration. We have previously observed similarly low levels of XMRV antibodies in a study of 40 PC patients [6] and in an expanded cohort of nearly 300 PC patients (unpublished). The results of our current study are also in line with those of a recent report of XMRV infection in a non-human primate model [40]. That study revealed a pattern of relatively low antibody induction following the initial XMRV infection, and showed that this was only modestly boosted by a second infection 158 days later. Moreover, the antibody titers in that study decreased after both the prime and boost infections using live XMRV. While there was clearly a deficiency in eliciting a durable antibody response, the roles(s) of (i) possible replicative deficiencies of the virus in these primate hosts [41], (ii) immunosuppressive activity of the viral proteins [37], [38], [39] and/or (iii) host restriction factors [42], [43] will require further investigation. Another recent study reported the induction of XMRV NAbs using Gairdner's shrew-mice (*Mus pahari*) that express a functional XPR1 receptor and support a productive XMRV infection [41]. Using this model may provide opportunities to evaluate XMRV with regard to its pathogenesis, replication and vaccine-induced protection from infection. Lastly, our study raises the possibility that low immunogenicity may be an intrinsic characteristic of XMRV, which could in part account for some of the reported discrepancies in detecting XMRV in various patient cohorts.

### Conclusions

In summary, binding and neutralizing antibody responses elicited by XMRV VLP vaccination in a mouse model were characterized. The antibody titers decreased rapidly after immunization, which may be an intrinsic feature of XMRV immunogenicity. The relatively low and gradually decreasing humoral immune responses we observed may in part explain the low titers of antibodies detected in PC patients and the discrepancies among reports of XMRV seroprevalence in different cohorts of patients.

## Methods

### Cells lines

HeLa, Du145, Du145-C7, 293-AD and 293T cells were grown in DMEM supplemented with 10% FBS (CellGro). The Du145-C7 cell line that produce infectious XMRV was provided by Dr. R.H. Silverman (The Cleveland Clinic, Cleveland, OH). TZM-bl cells were obtained from the National Institutes of Health AIDS Research and Reference Reagent Program (Catalog number 8129). TZM-bl cells express CD4 and co-receptors, CCR5 and CXCR4, for HIV-1 infection and contain integrated reporter genes for firefly luciferase and *Escherichia coli* β-galactosidase under control of an HIV-1 long-terminal repeat sequence [44], [45], [46]. The 293-AD cell line, derived from HEK293 cells with improved cell adherence and plaque forming properties, was purchased from Stratagene (Cat. No. 240085; La Jolla, CA, USA).

### Plasmids and recombinant Ad5 vectors

Codon optimized sequences of XMRV *gag* and *env* (GenBank accession numbers JF309078 and JF309077, respectively) were synthesized by GenScript Corporation (Piscataway, NJ) and cloned into pUC57 vector. The *env* sequence was then cloned into the first CMV-driven expression cassette of pDP1 Shuttle vector using AgeI and XbaI restriction enzymes, resulting in the plasmid pDP1-XMRV*env*. The XMRV *gag* gene was cloned into the second MCMV-driven expression cassette of the pDP1-XMRVenv using EcoRI and HindIII, resulting in pDP1-XMRV*envgag*. The details and complete sequences of pDP1, pDP1-XMRV*env* and pDP1-XMRV*envgag* are available upon request. Finally, pDP1-XMRV*envgag* was recombined with the pAdEasy-1 plasmid by co-transfection into 293-AD cells using Lipofectamine 2000 to produce the recombinant Ad5, Ad5-XMRV. The control Ad5 vector, Ad5-Luc, which expresses the luciferase gene was produced in a similar manner and described previously [47]. The recombinant Ad5 vectors were purified by double centrifugation on cesium chloride gradients and subjected to dialysis as described [48]. The physical titers, or total virus particles (VP), were determined spectrophotometrically by measuring the OD at 260 nm where 1 absorbance unit is equivalent to 1.1×10^12^ virus particles [49]. Viral titers were determined by a standard 50% tissue culture infectious dose (TCID_50_) assay using 293-AD cells. The TCID_50_ was converted to plaque forming units (PFU) per ml where the PFU/ml has been empirically determined to be 0.7 log less that the TCID_50_/ml.

### Production of HIV and XMRV pseudoviruses and XMRV VLP

Pseudoviruses were produced by co-transfecting into 293T cells the plasmid pSG3ΔEnv (AIDS Research and Reference Reagent Program; Catalog Number 11051; [46], [50]) with plasmids expressing either the HIV-1 or XMRV *env* (pDP1-XMRV*env*) gene. Virus containing cell media was collected after 48 hours of infection. Infectivity of XMRV and HIV-1 pseudoviruses was compared by detection of beta-galactosidase expression 48 hours after infection of TZM-bl cells.

To produce XMRV VLP, HeLa cells were infected (10 MOI) with Ad5-XMRV in DMEM with 2% FBS for 16 hours for virus absorption and then the media was replaced with fresh growth media. Culture media was then collected after 48 hours of infection, passed through a 0.45- µm filter (Whatman, Florham Park, NJ) and concentrated ∼1,000 times by ultracentrifugation at 25,000 xg through 20% sucrose in PBS buffer. Purified VLP were stored in aliquots after total protein concentration was detected and subsequently used for immunization of mice and for coating ELISA plates and immunoblotting.

### Immunization

For immunization, 10 Balb/c mice (Charles River) were first primed with DNA (25 µg of pDP1-XMRV plasmid per mouse in 50 µl of Saline) and then boosted 22 and 50 days later with Ad5-XMRV (2×10^9^ virus particles per mouse in 100 µl of Saline). Mice were then boosted again on Day 100 with XMRV VLP (7.5 µg per mouse in 50 µl of Saline). All immunizations were done intramuscularly in femoral muscle. The control mice were primed with the same amount of empty plasmid and boosted with adenoviruses expressing the beta-galactosidase gene.

### Neutralization assay

Monoclonal antibodies (mAb 83A25 and mAb b12; AIDS Reference and Reagent Program, Catalog No. 2640), polyclonal antibodies (Goat anti-Friend MLV, ATCC catalog # VR-1537AS-Gt) and mouse sera were assayed for the presence of neutralizing activity against XMRV pseudoviruses using a single-round pseudotype reporter assay described previously [19]. The monoclonal antibody mAb 83A25 was a kind gift from Dr. Leonard Evans (NIAID NIH, Rocky Mountain Laboratories). Briefly, TZM-bl cells were plated and cultured overnight. A total of 2,000 infectious units of pseudotyped virus were combined with fivefold dilutions of heat-inactivated test serum and incubated for 1 hour at 37°C. Noninfectious heat-inactivated mouse serum was added as necessary to maintain a constant overall concentration. The virus-antibody mixture was then added to TZM-bl cells, and after two days, the cells were lysed, and the luciferase activity of each well was measured using a luciferase assay reagent (Promega, Madison, WI) and a Synergy HT luminometer (Bio-Tek, Winooski, VT). Background luminescence was determined in uninfected wells and subtracted from all experimental wells. Cell viability and toxicity were monitored by basal levels of luciferase expression and by visual inspection. Relative neutralization (percentage of control) was calculated by dividing the number of luciferase units at each serum dilution by the values in wells containing no test serum and subtracting that value from the values in wells containing no test serum. The dilution of antibody or sera that neutralizes infection by 50% (NT50) was then calculated using the GROWTH function in Excel version 12.2.5.

### Immunoassays

#### XMRV ELISA

For detection of XMRV-specific antibodies in mouse sera, an indirect ELISA was performed. XMRV VLP (3 µg/ml) in CB2 buffer (Immunochemistry Technologies LLC, Bloomington, MN) was immobilized on Immunoplates (NAlgeNunc, Rochester, NY), according manufacturer protocol, and incubated with serial dilutions of mouse sera. Specific antibodies were detected with goat anti-mouse HRP-conjugated IgG (H+L) (Southern Biotech, Birmingham, AL) and OPD substrate (Thermo Science, Rockford, IL). Mouse polyclonal antibodies were purified from mouse sera using Nab Protein A/G Spin Kit (Thermo Scientific, Rockford, IL) that allows small-scale affinity purification of antibodies from serum. The endpoint titer is defined as the reciprocal of the highest dilution of a serum that gives a reading above the cutoff. Cutoff was calculated for each dilution using equation Cutoff  =  X*SD*f,* where X is average and SD is standard deviation values measured for control serum, and f is SD multiplier corresponding to the confidence level 95% and number of replicates [51].

#### HIV-1p24 ELISA

Extracellular p24 was measured using the Alliance HIV-1 p24 ELISA kit (Perkin-Elmer) according to the manufacturer's instructions. Cell-free supernatants from infected cultures were harvested and stored at -80°C prior to quantification.

### Immunoblotting

Immunoblotting was performed using ether cell lysate after infection with Ad5 vector or purified VLP. Samples were separated by 12% SDS-PAGE, transferred to Immun-Blot™ PVDF membrane (Bio-Rad, Hercules, CA), blocked with 5% BSA in TBS-T buffer (50 mM Tris, 150 mM NaCl, 0.05% Tween-20, pH 7.4) and probed with R187 anti-Gag monoclonal antibody [52] or 83A25 anti-Env monoclonal antibody [53]. R187 antibody has been shown to react with XRMV Gag [1], [4] and 83A25 antibody weakly recognizes a common epitope in SU of gamma-retroviral envelope proteins [53]. After incubation with secondary antibody, HRP-conjugated anti-rat IgG (Southern Biotech, Birmingham, AL), protein bands were visualized with enhanced chemiluminescence detection reagents (Amersham Pharmacia).

### Flow cytometry

For flow cytometry analysis of XMRV *env* gene expression HeLa cells infected with 10 MOI of adenoviral vector for 48 hours were permeabilized with Cytofix/Cytoperm (BD Bioscience) at 4°C for 20 min. After washing three times with Perm Wash Buffer (BD Bioscience), cells were incubated with 1∶10 dilution of mAb 83A25 [53] cell culture media at 4°C for 30 min. Cells were then washed again and incubated with 1∶200 diluted FITC-conjugated goat anti-rat IgG (Southern Biotech, Birmingham, AL) at 4°C for 30 min. Cells were washed and analyzed on FACSCalibur flow cytometer (BD Bioscience). Data were acquired with CellQuest software and analyzed with FlowJo version 8.8.6 software.

### Transmission electron microscopy (TEM) to detect XMRV and XMRV VLP

Transmission electron microscopy was performed at the Emory Robert P. Apkarian Integrated Electron Microscopy Core as described previously [54]. Approximately 10^6^ XMRV or XMRV VLP infected HeLa cells were pelleted, then treated as a cell block: fixed initially in 2.5% buffered glutaraldehyde, postfixed in 1% osmium tetroxide in the same buffer, and dehydrated through graded ethanol. The fixed cells were infiltrated with propylene oxide and embedded in Embed-812 (Electron Microscopy Sciences, Fort Washington, PA). Ultrathin sections (60–70 nM) were cut examined using an H-7500 transmission electron microscope (Hitachi, Pleasanton, CA).
