# Correction: Antibody Responses against Xenotropic Murine Leukemia Virus-Related Virus Envelope in a Murine Model

**DOI:** 10.1371/annotation/913fdc1e-877e-4c70-ac20-761d2d72400d

**Published:** 2011-05-04

**Authors:** Natalia Makarova, Chunxia Zhao, Yuanyuan Zhang, Sushma Bhosle, Suganthi Suppiah, Jeanne M. Rhea, Natalia Kozyr, Rebecca S. Arnold, Hinh Ly, Ross J. Molinaro, Tristram G. Parslow, Eric Hunter, Dennis Liotta, John Petros, Jerry L. Blackwell

Some potential competing interests in relation to this work were not declared in the article. The authors apologize for this omission and would like to disclose the following information: Drs Blackwell, Makarova, Molinaro, Suppiah and Ly have a U.S. patent (PCT/US2010/050633) and pending international patent application (WO 2011/041350 A2) on compositions and methods for analyzing and treating a disease, disorder, or condition associated with Xenotropic Murine Leukemia Virus-Related Virus infection. 

